# Short-duration, submaximal intensity exercise stress combined with adenosine triphosphate decreases artifacts in myocardial perfusion single-photon emission computed tomography

**DOI:** 10.1186/s12872-020-01720-w

**Published:** 2020-10-07

**Authors:** Yukinori Shinoda, Koichi Tachibana, Tomoko Minamisaka, Hidetada Fukuoka, Hirooki Inui, Keisuke Ueno, Soki Inoue, Kentaro Mine, Kumpei Ueda, Shiro Hoshida

**Affiliations:** Department of Cardiovascular Medicine, Yao Municipal Hospital, 1-3-1 Ryuge-cho, Yao, Osaka, 581-0069 Japan

**Keywords:** Adenosine triphosphate, Artifacts, End-diastolic volume, Exercise, Myocardial perfusion imaging, Rate pressure product

## Abstract

**Background:**

Myocardial perfusion single-photon emission computed tomography (SPECT) imaging with stress is a useful examination for detecting coronary artery disease. Since the presence of artifacts is remaining challenges, we aimed to define the minimum intensity of low-grade exercise stress levels combined with drug stress to reduce undesired artifacts and their related factors.

**Methods:**

We divided patients with suspicious coronary artery disease into 4 groups as follows: group A, adenosine triphosphate (ATP) for 6 min; group A + 25 W, ATP + 25 W exercise for 6 min; group A + 35 W, ATP + 35 W exercise for 6 min; group A + 45 W, ATP + 45 W exercise for 6 min) and enrolled only those whose summed stress scores were < 3. Undesired artifacts were evaluated on the basis of heart-to-liver activity (*H/L*) ratio and heart-to-10 pixels below the heart (*H*/below the *H*) ratio.

**Results:**

The logarithmic values of *H/L* and *H*/below the *H* ratios were significantly higher in groups A + 35 W and A + 45 W than in group A (*p* < 0.05, each). In all the patients, the logarithmic values of *H/L* and *H*/below the *H* ratios positively correlated with the increment of rate pressure product (RPP, *p* = 0.002 and *p* = 0.005, respectively) after stress in the univariate analysis. The left ventricular end-diastolic volume (LVEDV) after stress (*p* = 0.002) negatively correlated with the logarithmic value of *H*/below the *H* ratio, but not *H/L* ratio. Although the increment of RPP was independently associated with the logarithmic values of both *H/L* (*p* = 0.001) and *H*/below the *H* ratios (*p* = 0.005), LVEDV was also independently associated with the logarithmic value of *H*/below the *H* ratio (*p* < 0.001) in multivariate regression analysis under adjusting with age and sex.

**Conclusion:**

ATP plus ≥35 W exercise stress for 6 min was useful for reducing undesired artifacts after stress in myocardial perfusion SPECT. LVEDV after stress in addition to the increment of RPP was independently associated with the *H*/below the *H* ratio, but not the *H/L* ratio.

## Background

It is well known that myocardial single-photon emission computed tomography.

(SPECT) imaging is useful for detecting patients with significant myocardial ischemia.

These patients can then be assessed for coronary intervention therapy. Streak artifacts are the most common source of error in myocardial SPECT imaging. Stress with vasodilators has become increasingly used in myocardial perfusion SPECT, but high liver activity adjacent to the inferior wall results from oversubtraction of the activity from the inferior wall [1, 2]. Myocardial tracer uptake was greatest with administration of vasodilators, such as adenosine, and this effect was not attenuated by combining vasodilator administration with exercise [3]. Although adding low-level exercise stress to drug stress may help overcome some of the limitations of drug infusion alone and is useful for reducing the streak artifact [4–6], no direct comparison has been made between drug stress and the stress combined with bicycle exercise at various low grades. In this study, we hypothesized a certain grade, but not maximum grade, of bicycle exercise protocol is adequate to avoid streak artifacts when combining a 6-min infusion with the adenosine precursor, adenosine triphosphate (ATP), as compared with the standard ATP-only protocol. We evaluated the differences in the reduction of undesired artifacts between two different background regions, as well as the differences in the relevant clinical factors on myocardial perfusion SPECT in patients with suspicious coronary artery disease. We only included patients whose summed stress scores (SSSs) were < 3 for a robust and accurate evaluation of artifacts.

## Methods

Subjects with suspected angina pectoris who were unable to generate adequate stress during exercise because of antianginal medications or confounding factors that might have hindered them from achieving the target heart rate underwent elective 99mTc-tetrofosmin stress myocardial perfusion imaging (MPI). The patients (*n* = 280, from December 2015 to September 2017) were randomly allocated to one of the four stress protocols by an envelope method (*n* = 70 each) as follows: ATP [7, 8] at 120 μg/kg for 6 min (group A) and ATP at 120 μg/kg for 6 min concomitantly combined with various exercise intensities for 6 min at 25, 35, and 45 W (groups A + 25 W, A + 35 W, and A + 45 W, respectively). The subjects exercised on a bicycle ergometer and received 110 MBq of ^99m^Tc tetrofosmin injection at 3 min after initiation of ATP infusion. Image acquisition was initiated 45 min after the ^99m^Tc injection. A 2-min planar image was obtained, followed immediately by the acquisition of a standard gated SPECT image. Perfusion imaging was performed using a 2-detector gamma camera (ADAC Forte), with a circular 180°orbit. The perfusion data were displayed in a 17-segment model in accordance with the American Society of Nuclear Cardiology guidelines [9]. The 17 segments for each image were scored from 0 to 4, with 0 indicating no perfusion defect and 4 indicating no tracer. In this study, we enrolled only patients whose SSSs were < 3 because of robust clarification of artifacts. Therefore, we analyzed only the data of 109 patients (group A, *n* = 23; group A + 25 W, *n* = 34; group A + 35 W, *n* = 28; and group A + 45 W, *n* = 24). Whereas the stress and rest studies were interpreted together in the usual manner, the four-group images were analyzed in random order to avoid bias by the expert reader. The exclusion criteria were as follows: a contraindication to adenosine (moderate to severe chronic obstructive pulmonary disease or asthma, second- or third-degree atrioventricular block or sinus node disease, known as hypersensitivity to adenosine); hemodynamic instability; decompensated congestive heart failure; or the use of theophylline or dipyridamole within the preceding 48 h. Patient characteristics were examined in terms of laboratory data, medications, and comorbidities. Systolic and diastolic blood pressure and heart rate were recorded before and immediately after exercise or 6 min after ATP infusion. This study complied with the tenets of the Declaration of Helsinki, and all patients provided written informed consent to participate.

Undesired background artifacts were evaluated using two different modes. To obtain the heart-to-liver activity (*H/L*) ratio, the regions of interest were drawn around the heart and liver, excluding the gall bladder. “Heart counts” and “liver counts” were obtained from the point of maximum counts in each region of interest. Undesired artifacts were also evaluated on the basis of heart-to-10 pixels below the heart ratio (*H*/below the *H* ratio). The profile lines were placed on the heart and below the heart from the bottom of the heart image to 10 pixels. “Heart counts” were obtained from the maximum count in the profile line, and “below heart counts” were obtained from the minimum count in the profile line. These ratios were determined from the anterior planar images. The image readers were blinded to patient characteristics and group assignment.

Continuous variables are expressed as means ± standard deviations, whereas categorical variables are presented as percentages. Differences in categorical variables among the groups were assessed using chi-square tests (4 × 2), while those in continuous variables were assessed using one-way analysis of variance, and those between two groups were made using a post hoc Bonferroni test. The significance of the correlation between two variables was assessed with regression analysis. A *p* value of < 0.05 was considered statistically significant.

## Results

The clinical characteristics of the patients are shown in Table 1. No significant differences in age, sex, incidence of comorbidities, medications, and laboratory data except for low-density lipoprotein cholesterol levels were found among the four groups. The values of the hemodynamic parameters were significantly higher in the groups with concomitant exercise than in those without exercise (Table 2). Left ventricular end-diastolic volume (LVEDV), end-systolic volume, and ejection fraction were not significantly different among the four groups before and after the protocol.

**Table 1:** Patient characteristics

	Total	Group				***p*** Value
		A	A + 25 W	A + 35 W	A + 45 W	
	***n*** = 109	***n*** = 23	***n*** = 34	***n*** = 28	***n*** = 24	
**Age, years**	**70 ± 10**	**75 ± 9**	**68 ± 10**	**69 ± 10**	**71 ± 8**	**0.056**
**Male, %**	**41**	**48**	**32**	**39**	**50**	**0.508**
**Previous MI, %**	**4**	**13**	**3**	**0**	**0**	**0.051**
**Previous PCI, %**	**16**	**39**	**12**	**11**	**4**	**0.051**
**Cerebrovascular disease, %**	**17**	**31**	**7**	**14**	**25**	**0.071**
**Peripheral artery disease, %**	**8**	**22**	**3**	**7**	**4**	**0.061**
**Hypertension, %**	**62**	**73**	**65**	**54**	**58**	**0.479**
**Dyslipidemia, %**	**57**	**68**	**62**	**46**	**54**	**0.051**
**Diabetes mellitus, %**	**41**	**46**	**38**	**46**	**33**	**0.782**
**Atrial fibrillation, %**	**5**	**9**	**3**	**7**	**0**	**0.445**
**Current Smoking, %**	**15**	**23**	**15**	**11**	**13**	**0.715**
***Laboratory data***						
**Hb, g/dL**	**13.6 ± 1.7**	**12.7 ± 1.7**	**13.6 ± 1.4**	**14.1 ± 1.9**	**13.9 ± 1.8**	**0.051**
**HbA1c, %**	**7.8 ± 2.6**	**6.7 ± 1.2**	**8.2 ± 2.7**	**8.4 ± 3.2**	**7.4 ± 2.5**	**0.216**
**UA, mg/dL**	**5.4 ± 1.5**	**5.1 ± 0.8**	**5.2 ± 1.5**	**5.8 ± 1.8**	**5.6 ± 1.8**	**0.415**
**LDL-C, mg/dL**	**122 ± 35**	**108 ± 29**	**122 ± 30**	**139 ± 35**	**113 ± 41**	**0.036**
**HDL-C, mg/dL**	**54 ± 26**	**53 ± 15**	**61 ± 41**	**51 ± 10**	**48 ± 20**	**0.493**
**Creatinine, mg/dL**	**0.92 ± 0.64**	**1.23 ± 1.15**	**0.77 ± 0.21**	**0.81 ± 0.37**	**0.97 ± 0.45**	**0.054**
**BNP, pg/mL**	**49 (22, 78)**	**58 (22, 208)**	**48 (23, 68)**	**59 (15, 65)**	**40 (11, 63)**	**0.067**
***Medications***						
**ADA, %**	**22**	**14**	**27**	**29**	**17**	**0.167**
**ATA, %**	**22**	**36**	**27**	**11**	**17**	**0.192**
**β blocker, %**	**14**	**14**	**18**	**18**	**4**	**0.440**
**CCB, %**	**46**	**55**	**47**	**46**	**38**	**0.634**
**Diuretics, %**	**8**	**14**	**6**	**11**	**4**	**0.374**
**Nitrates, %**	**7**	**14**	**9**	**7**	**0**	**0.636**
**RAS-I, %**	**37**	**36**	**27**	**36**	**54**	**0.075**
**Statin, %**	**41**	**57**	**44**	**21**	**42**	**0.462**

Data are mean ± SD or percentage except for BNP level (median, 25–75%)

*MI* myocardial infarction, *PCI* percutaneous coronary intervention, *Hb* hemoglobin, *UA* uric acid, *LDL-C* low-density lipoprotein cholesterol; *HDL-C* high-density lipoprotein cholesterol, *BNP* brain natriuretic peptide, *ADA* antidiabetic agents, *ATA* antithrombotic agents, *CCB* calcium-channel blocker, *RAS-I* renin-angiotensin system inhibitor

**Table 2:** Hemodynamic and SPECT data

	Total	Group				***p*** Value
		A	A + 25 W	A + 35 W	A + 45 W	
***Hemodynamic parameters***						
**SBP pre, mmHg**	**144 ± 20**	**144 ± 21**	**148 ± 18**	**137 ± 22**	**148 ± 21**	**0.182**
**post, mmHg**	**178 ± 41**	**146 ± 26**	**187 ± 39**	**175 ± 40**	**202 ± 37**	**< 0.001**
**DBP pre, mmHg**	**76 ± 15**	**74 ± 15**	**77 ± 13**	**74 ± 16**	**80 ± 16**	**0.387**
**post, mmHg**	**80 ± 28**	**69 ± 19**	**77 ± 18**	**80 ± 38**	**95 ± 30**	**0.013**
**HR pre, bpm**	**76 ± 13**	**76 ± 12**	**75 ± 12**	**76 ± 17**	**78 ± 10**	**0.905**
**post, bpm**	**105 ± 22**	**80 ± 12**	**107 ± 18**	**105 ± 17**	**124 ± 17**	**< 0.001**
**RPP pre**	**10,902 ± 2282**	**10,854 ± 2236**	**11,054 ± 2296**	**10,266 ± 2260**	**11,476 ± 2241**	**0.28**
**post**	**19,109 ± 6880**	**11,690 ± 2916**	**20,089 ± 5625**	**18,377 ± 4741**	**25,685 ± 6467**	**< 0.001**
**delta**	**8207 ± 6250**	**836 ± 2265**	**9035 ± 4769**	**8111 ± 3554**	**14,209 ± 6173**	**< 0.001**
***SPECT data***						
**EDV stress, mL**	**67 ± 23**	**64 ± 24**	**72 ± 26**	**66 ± 23**	**65 ± 16**	**0.505**
**rest, mL**	**68 ± 23**	**65 ± 24**	**71 ± 26**	**68 ± 23**	**66 ± 18**	**0.721**
**ESV stress, mL**	**21 ± 13**	**21 ± 12**	**24 ± 16**	**21 ± 11**	**18 ± 9**	**0.46**
**rest, mL**	**20 ± 13**	**20 ± 12**	**20 ± 13**	**20 ± 11**	**17 ± 9**	**0.672**
**EF stress, %**	**70 ± 10**	**68 ± 11**	**70 ± 12**	**69 ± 8**	**73 ± 7**	**0.286**
**rest, %**	**73 ± 9**	**71 ± 9**	**73 ± 9**	**72 ± 9**	**76 ± 8**	**0.286**
**SSS**	**1.3 ± 1.2**	**1.0 ± 1.2**	**1.2 ± 1.1**	**1.6 ± 1.1**	**1.4 ± 1.2**	**0.345**
**SRS**	**1.3 ± 1.6**	**1.0 ± 21.9**	**1.1 ± 1.3**	**2 ± 1.7**	**1.1 ± 1.2**	**0.06**
**SDS**	**0.7 ± 0.9**	**0.7 ± 1.1**	**0.7 ± 0.8**	**0.6 ± 0.6**	**0.9 ± 1.1**	**0.685**

Data are mean ± SD

*SBP* systolic blood pressure, *DBP* diastolic blood pressure, *HR* heart rate, *RPP* rate pressure product, *EDV* end-diastolic volume, *ESV* end-systolic volume, *EF* ejection fraction, *SSS* summed stress score, *SRS* summed rest score, *SDS* summed difference score

**Fig. 1:**
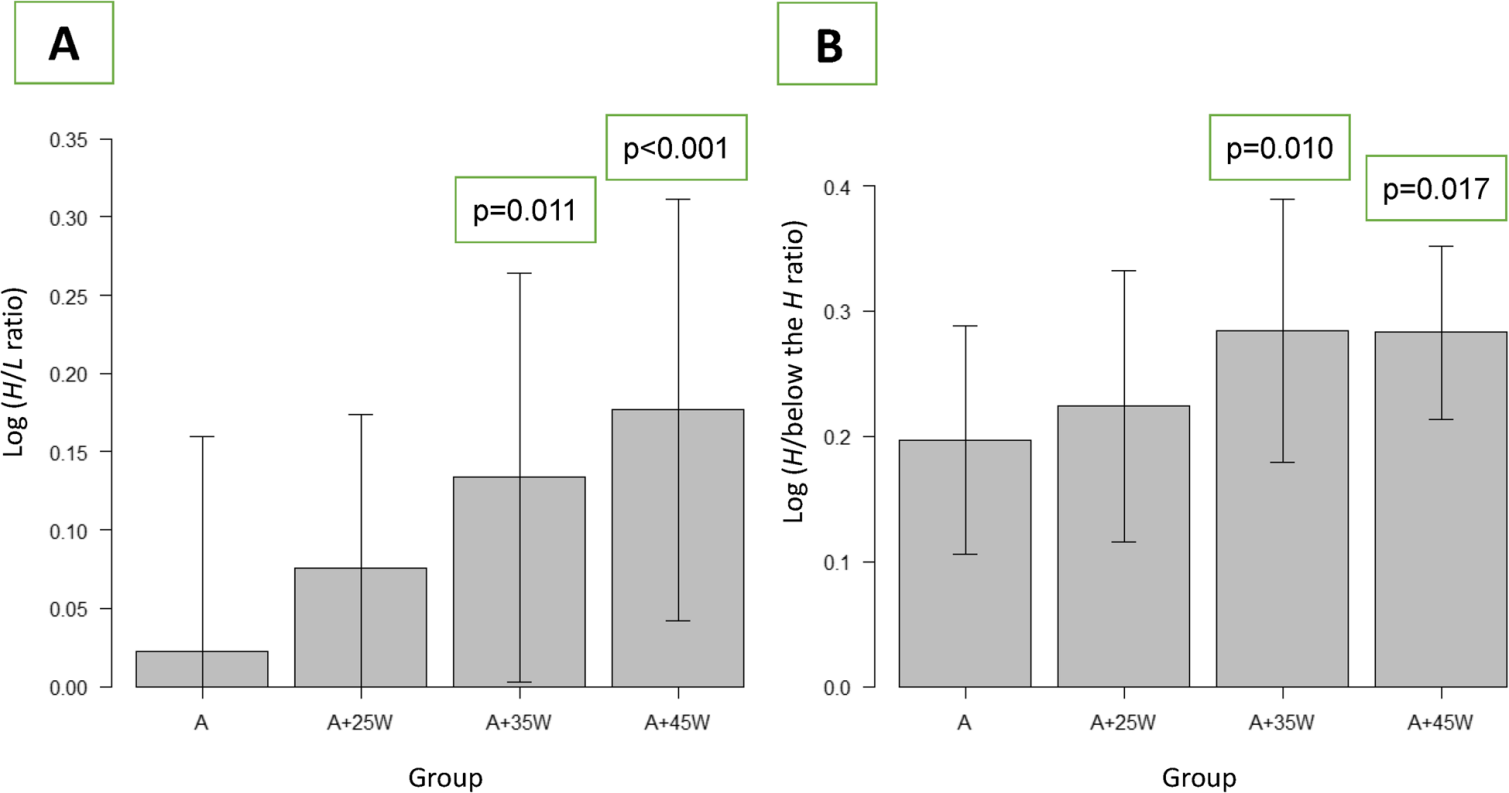
Differences in the logarithmic values of the heart-to-liver activity (*H/L*) ratio (ANOVA, *p* < 0.001) (**a**) and heart-to-below the heart activity (*H*/below the *H*) ratio (ANOVA, *p* = 0.002) (**b**) among the four groups (mean ± SD). The values calculated using the post hoc Bonferroni test in groups A + 35 W and A + 45 W were significantly different compared with those in group A. *P* values are indicated as compared with group A

In terms of SPECT data, maximum signals from the heart were slightly decreased after each exercise protocol. In contrast, maximum signals from the liver and minimum signals below the heart were significantly reduced in association with exercise (ANOVA, *p* = 0.010 and *p* = 0.045, respectively). The logarithmic value of the heart-to-background count ratios such as the *H/L* ratio and *H*/below the *H* ratio was significantly higher in groups A + 35 W and A + 45 W than in group A (Fig. 1).

In all the patients, the logarithmic values of *H/L* and *H*/below the *H* ratios positively correlated with heart rate (*p* = 0.004 and *p* = 0.008, respectively), rate pressure product (RPP; *p* = 0.005 and p = 0.008, respectively), and the increment of RPP (delta RPP; *p* = 0.002 and p = 0.005, respectively) after stress in the univariate analysis (Table 3). The LVEDV after stress (p = 0.002) negatively correlated with the logarithmic value of *H*/below the *H* ratio, but not *H/L* ratio. Although male sex was independently associated with the logarithmic values of both *H/L* ratio (*p* = 0.020) and *H*/below the *H* ratio (*p* = 0.001) in the multivariate regression analysis, LVEDV was also independently associated with the logarithmic value of *H*/below the *H* ratio (*p* < 0.001; Table 3).

**Table 3:** Correlation with heart-to-background activity ratio

	Univariate		Multivariate	
	***r*** Value	***p*** Value	***p*** Value	Coefficient (95% CI)
**log (*H*/*L*** **ratio)**				
**Age**	**0.030**	**0.752**	**0.603**	**0.0006 (− 0.0018 ~ 0.0032)**
**Male**	**0.196**	**0.040**	**0.020**	**0.0591 (0.0093 ~ 0.1089)**
**delta RPP**	**0.283**	**0.002**	**0.001**	**0.0001 (0.0001 ~ 0.0001)**
**log (*H*/below the** ***H*** **ratio)**				
**Age**	**0.027**	**0.773**	**0.686**	**−0.0003 (− 0.0023 ~ 0.0015)**
**Male**	**0.126**	**0.188**	**0.001**	**0.0677 (0.0275 ~ 0.1079)**
**delta RPP**	**0.263**	**0.005**	**0.005**	**0.0001 (0.0001 ~ 0.0001)**
**EDVex**	**0.289**	**0.002**	**< 0.001**	**−0.0019 (− 0.0028 ~ − 0.0010)**

*CI* Confidence interval, *H/L* heart-to-liver activity ratio, *H/below the H* heart-to-below the heart, *RPP* rate pressure product, *EDVex* end-diastolic volume after exercise

No significant side effects except for mild nausea and hypotension attributable to the ATP infusion in combination with exercise were observed, and our protocol was well tolerated in this study.

## Discussion

The main finding of our study was that concomitant 6-min low-grade exercise supplementation with ≥35 W, through the use of the bicycle protocol, increased heart-to-background count ratios such as *H/L* and *H*/below the *H* ratios in the patients who underwent ATP stress MPI. The other findings of this study were that in the case of the *H*/below the *H* ratio, the artifact was independently associated with the LVEDV after exercise in addition to the increment of RPP.

Exercise stress is well known to increase heart-to-background count ratios as compared with drug stress alone and to reduce hepatic tracer uptake relative to the heart. Side effects are common during standard adenosine stress testing. However, few significant side effects attributable to the ATP infusion in combination with exercise were observed, and this procedure was well tolerated in this study. It is conceivable that exercise increases sympathetic nerve activity, which improves atrioventricular conduction. Therefore, noncardiac side effects such as hypotension, and major arrhythmias such as atrioventricular block are significantly reduced after exercise stress [10]. Exercise also suppresses the vasodilation of adenosine in diaphragmatic regions and the heart-to-background count ratio is higher in the exercise groups in correlation with the exercise level achieved [3]. Samady et al. found that a 6-min adenosine infusion with concomitant low-level treadmill exercise reduced unfavorable side effects, enhanced image quality, and may have resulted in greater detection of ischemia [11]. Enhancement of image quality was also observed when the adenosine infusion study was performed as compared with the bicycle-exercise protocol in patients treated with beta-blockade [12]. However, the opposite results were reported by Jamil et al., who showed no change in defect size or severity following exercise, compared with the standard 6-min adenosine infusion [13].

A clear increase in *H/L* ratio is an important finding because an increased *H/L* ratio has been shown to result in fewer artifacts in the inferior and inferoseptal regions of the heart [1, 2]. In our study, we included only patients whose SSSs were < 3 to evaluate precisely the extent of background level. When combined with ATP plus short-duration exercise stress, mild stress was adequate to obtain a significant quality image. Some differences were found between the results in the two regions regarding the quantification of the target-to-background ratio. The *H*/below the *H* ratio represents a comparison of the image quality of the heart with those of organs adjacent to the heart, such as the diaphragm, intestine, and other digestive tissues, and was inversely dependent on LVEDV in association with the increment of RPP. Enlargement of the left ventricle may reduce the sharpness of the SPECT image because of the increase in the dispersion of ^99m^Tc tetrofosmin in case of *H*/below the *H* ratio. By contrast, male sex, irrespective of left ventricular size, was an independent factor for *H/L* or *H*/below the *H* ratio. Sex-related differences in artifacts may result from the grade of fat tissue and complicated body structures, including the lung, spine, and chest wall. A pertinent question that should be answered in the future is how a higher image quality can be obtained in patients with enlarged left ventricles when the use of a new type of stress method is becoming widespread in myocardial perfusion SPECT.

A considerable interpatient variability exists in the heart-to-background count ratios, but some extent of the exercise level achieved greater heart-to-background count ratio, which may depend on the extent of the RPP. A short-duration exercise stress of ≥35 W may enhance sympathetic activity abruptly and adequately, thus leading to the desirable results. Improvement of the heart-to-background count ratio was present at the lower exercise levels, which most patients could be expected to achieve. Six-min ATP infusion with a moderately low-grade exercise protocol during the same period is preferable because it can be incorporated into the standard drug stress regimen with no time loss.

## Limitations

Depending on the protocol design, the type of tracer, moment of tracer injection, and type and duration of exercise may vary [14–18] and elicit different results. Whether these issues have any effects on the accuracy of the perfusion study are currently unclear. We could not use SPECT-computed tomography scanner to reduce artifacts. As we randomly assigned patients into four groups but enrolled only those with SSSs of < 3, the number of patients differed among the groups. Under this condition, the defect severity and sensitivity to coronary artery disease in each group could not be evaluated.

## Conclusion

ATP administration concomitant with mild exercise stress of ≥35 W was useful for reducing undesired artifacts evaluated on the basis of heart-to-background ratio. *H*/below the *H* ratio, but not *H*/*L* ratio, was associated with the LVEDV after stress in addition to male sex and the increment of RPP. The factors that cause artifacts might be different between target organs.

## Abbreviations

ATP: Adenosine triphosphate
H: Heart
L: Liver
LVEDV: Left ventricular end-diastolic volume
MPI: Myocardial perfusion imaging
RPP: Rate pressure product
SPECT: Single-photon emission computed tomography
SSS: Summed stress scores
